# A CT method for following patients with both prosthetic replacement and implanted tantalum beads: preliminary analysis with a pelvic model and in seven patients

**DOI:** 10.1186/s13018-016-0360-7

**Published:** 2016-02-24

**Authors:** Henrik Olivecrona, Gerald Q Maguire, Marilyn E. Noz, Michael P. Zeleznik, Uldis Kesteris, Lars Weidenhielm

**Affiliations:** Department of Molecular Medicine and Surgery, Karolinska Institutet, SE-17176 Stockholm, Sweden; School of Information and Communication Technology, KTH Royal Institute of Technology, Stockholm, Sweden; Department of Radiology, New York University School of Medicine, 560 First Avenue, New York, NY 10016 USA; School of Computing, College of Engineering, University of Utah, Salt Lake City, UT USA; Department of Orthopedics, Skåne University Hospital, Lund, Sweden

**Keywords:** Radiostereometry, Longitudinal studies, CT analysis, RSA

## Abstract

**Background:**

Radiostereometric analysis (RSA) is often used for evaluating implanted devices over time. Following patients who have had tantalum beads implanted as markers in conjunction with joint replacements is important for longitudinal evaluation of these patients and for those with similar implants. As doing traditional RSA imaging is exacting and limited to specialized centers, it is important to consider alternative techniques for this ongoing evaluation. This paper studies the use of computed tomography (CT) to evaluate over time tantalum beads which have been implanted as markers.

**Methods:**

The project uses both a hip model implanted with tantalum beads, acquired in several orientations, at two different CT energy levels, and a cohort of seven patients. The model was evaluated twice by the same observer with a 1-week interval. All CT volumes were analyzed using a semi-automated 3D volume fusion (spatial registration) tool which provides landmark-based fusion of two volumes, registering a target volume with a reference volume using a rigid body 3D algorithm. The mean registration errors as well as the accuracy and repeatability of the method were evaluated.

**Results:**

The mean registration error, maximum value of repeatability, and accuracy for the relative movement in the model were 0.16 mm, 0.02° and 0.1 mm, and 0.36° and 0.13 mm for 120 kVp and 0.21 mm, 0.04° and 0.01 mm, and 0.39° and 0.12 mm for 100 kVp. For the patients, the mean registration errors per patient ranged from 0.08 to 0.35 mm. These results are comparable to those in typical clinical RSA trials. This technique successfully evaluated two patients who would have been lost from the cohort if only RSA were used.

**Conclusions:**

The proposed technique can be used to evaluate patients with tantalum beads over time *without* the need for stereoradiographs. Further, the effective dose associated with CT is decreasing.

## Background

To date, radiostereometric analysis (RSA) is considered the gold standard [1, 2] for precise monitoring of small movements of orthopedic joint implants. This monitoring is important because small movements of the implant early after implantation give an early indication of an increased risk for aseptic loosening and subsequent revision [3]. It has thus become important for pre-market evaluation of new joint implants as well as long-term evaluation of patients. Since the introduction of implant registries in Sweden, the cohort of patients with tantalum beads is approximately 10,000. More than half a million patients with similar prosthetic replacements have not had tantalum beads implanted; hence, they cannot be evaluated using RSA [4, 5]. Moreover, traditional RSA imaging must be performed very exactly [6], as it requires that the patient be positioned precisely with respect to a calibration cage and two X-ray tubes which must be used simultaneously to produce a usable steroradiograph. This radiograph must include a sufficient number of fixed tantalum beads (i.e., tantalum beads which have not moved) in the patient, which have a fixed and reproducible relation to the markers in the cage so that the examination can be correctly duplicated over time. Frequent repeats of an examination are necessary to fulfill these requirements. Furthermore, special training must be provided as well as special software written or purchased to evaluate the examination. Hence, it is important to consider alternative techniques for following *all* of these patients over time.

Development of computed tomography (CT) imaging technology has been rapid during the last decades. Currently, CT routinely provides high-resolution volume data with voxels of sub-millimeter size in all dimensions. Therefore, the small tantalum beads implanted as RSA markers can be detected in CT volumes with reasonable accuracy, and then this data can be used as input for an analysis similar to that using the classic marker-based RSA system. Additionally, the effective radiation dose from CT is decreasing and has the potential to soon be comparable to that of the (minimally) two simultaneous X-ray images required by RSA.

Over the last decade, our research group has developed and reported on CT measurement of orthopedic implants regarding position, wear, and migration *without* the requirement of marker implantation at surgery [7–14]. For this, we have developed and refined image post-processing tools that address these issues. This report explores this possibility of utilizing CT scans of patients implanted with tantalum beads for longitudinal evaluation by applying the proposed CT technique to (1) repeated scans of a pelvic model in varied orientations and (2) serial CT scans from a small patient cohort to test clinical applicability.

## Methods

We used both a pelvic model and a cohort of seven patients in this trial. These two studies are described below.

### Model

A plastic model of a human pelvis (Sawbones, Vashon, WA, USA) was implanted with 1-mm diameter tantalum beads using the same procedure as during marking of a patient during surgery and with a distribution of beads intended to simulate the typical marker configuration in hip arthroplasty patients to be followed with RSA, i.e., with beads placed in the periacetabular bone approached from within the acetabular fossa. Two uncemented acetabular cups were implanted in the model after each had been marked using eight tantalum beads in a circular fashion in the periphery of the opening of the polyethylene liner. In order to create more artifacts (i.e., a more realistic model), two different cups were used, and we would later choose the most difficult cup for analysis. The right side was a Trident cup with a 28-mm chrome-cobalt head (Stryker, Kalamazoo, MI, USA), while the left side was a Harris-Galante generation one (HG 1) cup with a 32-mm chrome-cobalt head (Zimmer, Warsaw, IN, USA).

A clinical CT scanner (Toshiba Aquilion ONE, Toshiba Medical Systems, Tochigi-ken, Japan) was used to acquire CT scans of the model in 25 different positions (to simulate different patient positions), where the model was lifted from the CT bed and repositioned in a new spatial orientation each time. For each position, two scans were obtained, one at a tube tension of 100 kV and one at 120 kV, producing two model volumes (representing lower versus higher radiation levels). All volumes were acquired using the same initial scout view; thus, the actual origin point in each volume with respect to the CT scanner was changed after each set of acquisitions. A 16-cm segment centered on the cups in the pelvic model was acquired in volume mode by scanning the entire segment with a single rotation with a revolution time of 0.275 s, an X-ray tube current of 80 mA, and an exposure time of 0.275 s resulting in 22 mAs. The volumes were reconstructed using a bone (convolution kernel FC35) algorithm into 320 slices at 0.5-mm increments with a matrix size of 512 × 512 giving a pixel resolution of 0.72 mm in *x* and *y*. The single-energy metal artifact reduction (SEMAR) algorithm was applied to each volume after reconstruction.

To simulate a patient “study,” any two of the 25 model volumes (at the same kilovoltage peak (kVp)) can be paired to represent the first and second patient volumes, yielding a unique spatial mismatch representing different patient positions in the scanner in a clinical situation but with zero change in the position of the prosthetic implant relative to the pelvis. We created 105 such “studies” by using all pairwise combinations (permutations) of 15 of the 25 volumes acquired at the same kVp. We chose only 15 volumes because it was thought that 105 pairs which created 105 studies were enough to show the potential of this method.

When visualizing small metal elements, even with the SEMAR algorithm applied, some beam hardening artifacts and partial volume effects can be expected and are indeed present. These deform the visual appearance of the tantalum beads and are most noticeable when a tantalum bead is in close proximity to the cup and located in the beam hardening tract. To *maximize* our estimates of the errors that the proposed technique would introduce, the cup side that appeared to have the *most* deformed markers was chosen for analysis, in all cases the left side.

### Patients

Seven patients who had undergone uncemented total hip arthroplasty were included. Approval from the Ethics Committee of the Lund University Dnr 2012/260 was obtained, and all patients gave their written consent. Four of these patients were randomly chosen from a pilot series before the start of a prospective 2-year longitudinal study of the Fitmore femoral stem (Zimmer, Warsaw, IN, USA). Three of the patients were randomly chosen from the cohort of patients in this Fitmore study group. For all patients, at surgery, the femoral stems were marked with tantalum beads in the extraction hole at the prosthetic collar and at the tip. These markers were attached using bone cement (Refobacin, Biomet, Warsaw, IN, USA) since they could not be inserted directly into the prosthesis due to restrictions by the commercial provider. As a part of this prospective multi-center study, these patients were followed using CT examinations (either on the same or different days) and were thus scanned using different CT units, with low-dose protocols, with different “types” of scans, and with different values of effective dose as seen in Table 1. This demonstrates that our method can be applied to a wide variety of patients with different clinical conditions. From Table 1, it is seen that three patients had studies separated by several days, two underwent a “double examination” as in RSA studies, and two with clinical and/or radiological symptoms that could be consistent with stem loosening had a “provocation study.” This last, which has been a part of clinical routine at the Karolinska University Hospital Solna for a decade [12] is one in which a pair of CT examinations were acquired in sequence: the first with forced internal rotation of the leg and the second with forced external rotation of the leg.

**Table 1:** Summary of patient studies. In some cases, the information necessary to calculate the effective dose was not available

Patient number	Study type	Pixel size, first scan (mm)	Slice spacing, first scan (mm)	Pixel size, second scan (mm)	Slice spacing, second scan (mm)	Effective radiation dose (mSv)
1	Provocation	0.23	0.5	0.23	0.5	NA
2	Double examination	0.81	0.5	0.81	0.5	4.1–3.2
3	21 days apart	0.33	1.0	0.37	1.0	2.4–3.4
4	3 days apart	0.24	0.5	0.24	0.5	NA
5	Provocation	0.78	0.5	0.78	0.5	5.4–5.4
6	Double examination	0.32	0.5	0.32	0.5	NA
7	12 days apart	0.29	0.5	0.37	1.0	6.6 (second scan only)

### Image analysis

All CT volumes were analyzed using a 3D volume fusion (spatial registration) tool [7, 15, 16]. This semi-automated tool provides landmark-based fusion of two volumes, registering a target volume with a reference volume via a variety of 3D transform modules, ranging from a simple rigid body to 3D warping and to user-defined polynomials. A graphical user interface provides numerous 3D and 2D analysis tools, including tools to visualize structures and designate landmarks while viewing from arbitrary positions, with simultaneous display of both reference and target volume information. For this study, the computer’s 3D pointing device was used to designate landmarks on 3D isosurfaces, with the software automatically finding the corresponding 3D points. A technical description can be found in earlier publications [8, 12, 17]. The procedures for analyzing the kinematics are described below.

### Manual processing

**Fig. 1:**
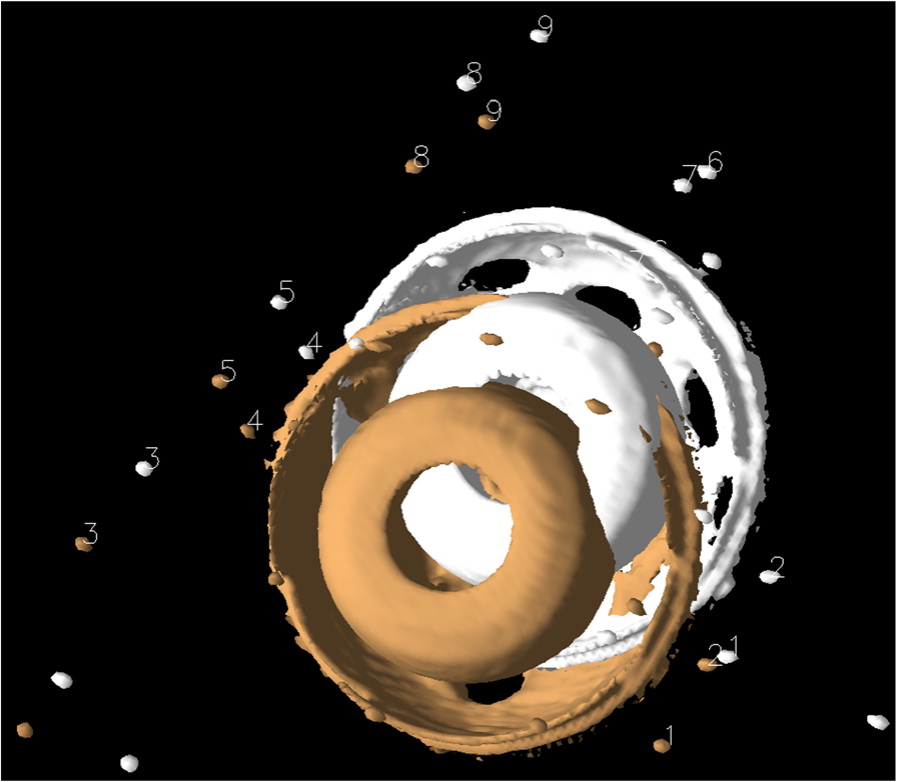
Model before registration—3D display. Landmark sequence numbers assigned to the reference and target volume tantalum beads implanted in the periacetabular skeleton. Isosurface display shows only the highly attenuating metal in the volumes

**Fig. 2:**
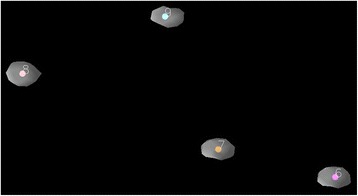
Close-up view of tantalum beads and associated landmarks. Semi-transparent display shows the computed landmarks to be embedded in the marker. Note deformity caused by partial volume effect

For each model study, we first visualized the tantalum beads as 3D isosurfaces simultaneously in both the first and second CT volumes, designated as the reference and target volumes, respectively. With the isosurface level set so that voxels less attenuating than metal were not visible, a 3D surface identifying the metal itself was obtained (see Fig. 1). For both volumes, a single preliminary landmark was manually designated on each tantalum bead, nine in the periacetabular skeleton (which we termed the “bone landmark set”) and eight in the polyethylene liner of the acetabular cup (which we termed the “prosthetic landmark set”), in a standardized order (see Fig. 1). For each preliminary landmark, the program automatically finds a best-fit center of the tantalum bead and records this as the final landmark point for that bead as described in [18] and shown in Figs. 1 and 2. For each prosthetic landmark set, the fusion tool automatically generated an additional “out of plane landmark” from a vector cross product to provide a consistent orientation and avoid the possibility of a mirror inversion during a subsequent rigid body transformation. Thus, both landmark sets now have nine landmarks. For each model study, the landmark sets were chosen twice by the same observer 1 week apart, thus forming two repeated trials.

For each patient study, landmarks were designated in the same way as for the model studies with regard to the tantalum beads that were visible in the femoral bone. Additionally, for the femoral component, landmarks were designated as the center of the head and on the tantalum bead in the extraction hole and at the distal tip. As with the model studies, for each prosthetic landmark set, the fusion tool automatically generated an additional out of plane landmark from a vector cross product. In two patients, the marker at the extractor hole could not be used since it was missing in one patient and a grossly moved in the other. In these patients, multiple surface points were marked on the entrance of the extraction hole, and the program automatically found a best-fit center as described in [14]. The femoral head landmark was designated by using a spherical landmark tool as previously described [18]. Landmarks were chosen in the patient volumes only once.

### Automated processing

The subsequent automated steps consisted of (1) registering the target volume to the reference volume using a rigid body transformation derived from the bone landmark sets, (2) transforming the target volume prosthetic landmark set into the reference volume coordinate system using the same rigid body transformation, (3) transforming both volumes and associated prosthetic landmarks from the reference coordinate system into a standard pelvic orientation coordinate system (where the pelvis has a fixed coronal orientation known as the McKibbin plane which includes the right and left spina iliaca anterior superior and the public tubercles), and finally (4) computing the rigid body transformation that would move the target prosthetic landmark sets into spatial alignment with the reference prosthetic landmark set [17]. Note that in (3) above, if no standard orientation rotation matrix had been associated with the reference volume, the identity matrix was applied. The rotation point for the last rigid body transformation is by default the centroid (the geometric weight point) of the reference prosthetic landmark set. The program computes all the rigid body transformations as a 3 × 3 rotation matrix and a 1 × 3 translation matrix. The registration method used is the same singular value decomposition (SVD) described by Söderkvist and Wedin [19].

**Fig. 3:**
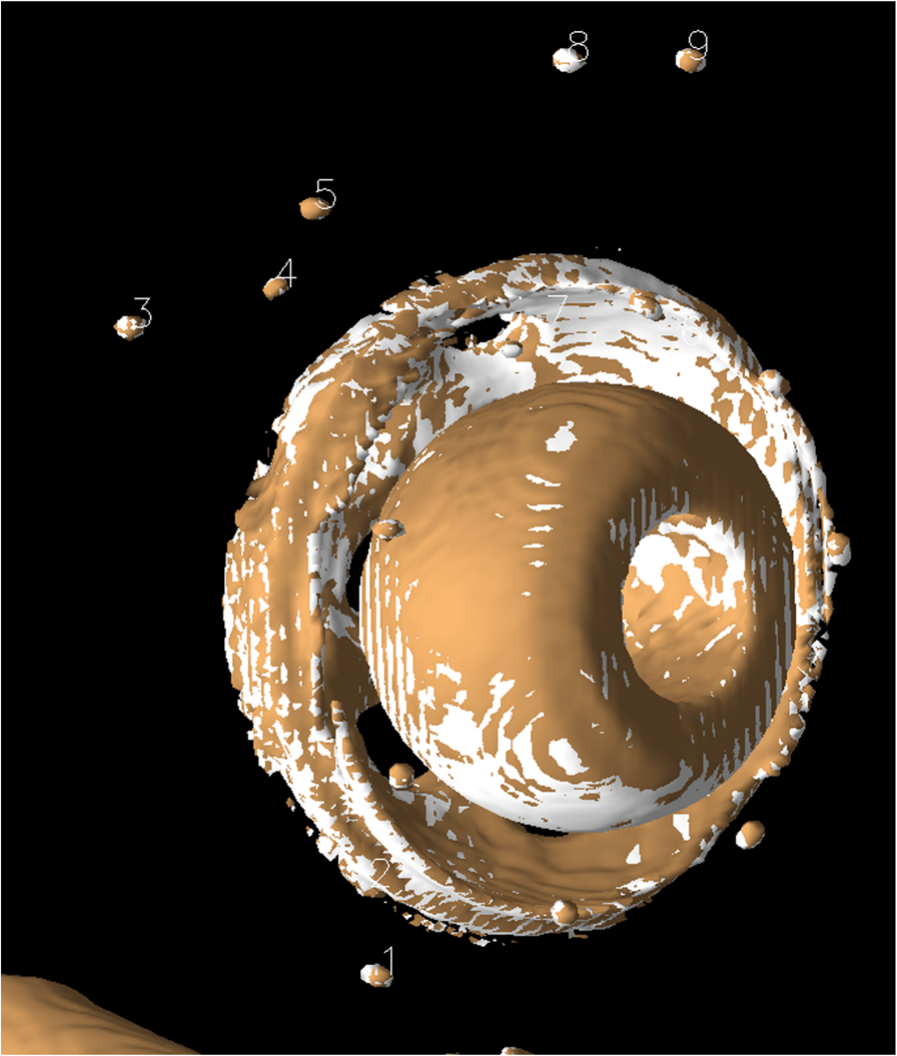
Model after registration of the markers corresponding to bone. In this experiment, the relative movement of the cup is zero. The overlapping pattern between the two examinations indicates that the surface representations are closer than the smallest voxel elements

The main results from this process were (1) visual 2D and 3D images (Fig. 3) of the two volumes after registration of the bone landmark set and (2) numerical data indicating the movement of the prosthesis in six degrees of freedom (rotations and translations along the *x*, *y*, and *z* axes, where the translations are given for each individual prosthetic landmark relative to the centroid of the prosthetic landmarks). The Euler angles, although not unique, were computed as a clockwise rotation first around the *x*, then the *y*, and finally the *z* principle axes. In addition, statistical information is generated about the rigid body transformation, expressed as distance differences between the original landmarks of the reference body and the new (transformed) locations of the target body for each landmark point, for both the bony and the prosthetic landmarks. These differences were generated for each orthogonal direction, in the *x*-*y* plane and for the volume. If the registration was perfect, these differences would be zero.

**Fig. 4:**
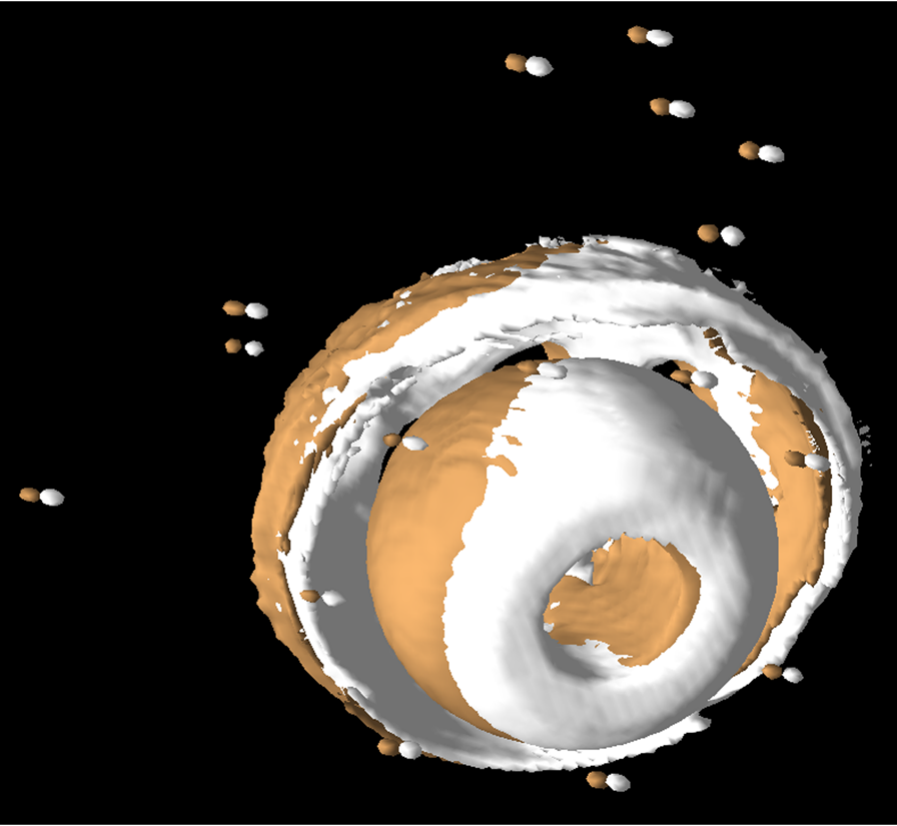
Model displayed after registration. A 2-mm displacement laterally has been introduced. Note how the tantalum beads and cup no longer overlap

In each case, the registered volumes were visually reviewed in 2D and in 3D to qualitatively validate the results. This is typically an interactive and very dynamic process, including rotating and zooming, changing 3D surface properties (e.g., solid, wire frame, dots, opacity, color), and changing isosurface parameters to shrink or grow surfaces, which provides numerous visual clues that cannot be conveyed in static images for publication. To demonstrate this, Fig. 4 shows one of the 120 kVp registered target volumes translated 2 mm relative to the original reference volume and viewed in Fig. 3. For a more detailed description of this method, see our previous study [17].

### Evaluation of errors

The model study error evaluation was divided into three parts (datasets):

1. Calculation of the distance difference between corresponding original target landmarks and reference landmarks across trials
2. Calculation of the distance difference between reference bone landmarks and transformed target bone landmarks after registration of the target landmarks
3. Relative movement of prosthesis after the final transformation expressed in six degrees of freedom (Euler angles and translation distances)

Ideally, all entries for the second and third datasets should be zero. To evaluate if parametric statistical methods could be used, these datasets were tested to see if they followed the normal (Gaussian) distribution using histograms, box, density, and quantile-quantile plots. Valstar et al. [6] have suggested that the accuracy and precision (repeatability) of RSA should be presented with the mean, median, and 95 % confidence interval (CI) and that RSA reports should quote all of these outcomes for each test. This was done for each dataset.

Repeatability of measurement of relative motion in the model was calculated as described by Bland and Altman [20, 21] and by Bragdon [22]. The repeatability limit is defined as the value less than or equal to the absolute difference between two test results obtained under repeatability conditions that may be expected to have a probability of 95 %, *assuming* a normal distribution of error [23]. Accuracy was evaluated as the distance of the data from the true value (in this case zero) as described by Ranstam [23]. Following Bragdon [22], an ANOVA was calculated over the 420 studies and individually over the 210 studies at 100 and 120 kVp to ensure that there was no difference within and between the studies [21].

Movement can be very ill-determined if the landmarks are badly configured. The same condition number presented by Söderkvist and Wedin [24] and used in RSA was calculated for the bone and prosthetic landmark sets. The condition number depends on the configuration of the landmarks and indicates whether the landmarks are distributed in a suitable way [24]. All of the evaluation of error calculations were performed using R version 3 [25].

## Results

We divide the results into those associated with the measurements of the model and those derived from the patient studies.

### Model

**Fig. 5:**
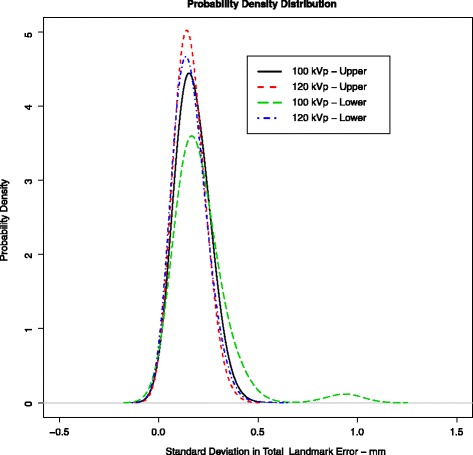
Density plot of errors of rigid body fitting for the first trial showing the Gaussian probability density or population frequency of the data. The plots are close to normal, but a skew in the tail of prosthetic landmarks at 100 kVp can be seen

In the model, all the nine markers in the bone and all eight markers in the cup could be visualized and designated with landmarks. The landmark designation procedure was rapid and required less than 5 min per volume. Since the 3D volumes could be freely rotated and viewed from arbitrary angles, it was easy to differentiate between tantalum markers. Before registration, there was spatial misalignment between all volumes (e.g., Fig. 1). After registration of the bone landmarks, all tantalum beads showed an overlapping pattern (e.g., Fig. 3). Numerically, the mean 3D-difference in individual landmarks in the original and registered volumes between repeated trials was 0.16 mm (range 0.01-0.51 mm, SD ±0.07 mm) for the 120-kVp series and 0.20 mm (range 0.02-1.06 mm, SD ±0.11 mm) for the 100-kVp series. This demonstrates that the effect of user input in this system is minimal and that there was only a small increase in the standard deviation when using the lower-dose CT scan (i.e., the scan at 100 kVp). The mean, range, standard deviation, and 95 % confidence interval for the landmark errors are given in Table 2. We note that the mean error for the prosthetic landmark group at 100 kVp was larger than for every other group. An analysis of the prosthetic landmark groups showed that at 100 kVp, the mean error for an individual landmark was between 0.11 and 0.14 mm (SD ±0.08-0.10 mm) except for landmark number five which had a mean error of 0.25 mm (SD ±0.25 mm). This is shown in the density plot given in Fig. 5 where the tail on the right of the density curve was longer for this 100-kVp prosthetic group. Visually, this tantalum bead was confirmed to be more distorted than the others and located in the beam hardening path of the acetabular shell. Examining the landmark registration errors for each individual landmark pair, we noted that each recorded registration error is much smaller than the shortest distance between any of the tantalum beads in the volumes, indicating that no landmarks were accidentally interchanged during the marking procedure.

**Table 2:** Mean landmark registration errors in the model (*n* = 945)

Volume	Mean (mm)	Range (mm)	Standard deviation (±mm)	95 % confidence interval (mm)
120-kVp markers in model bone	0.16	0.02–0.4	0.07	0.15–0.16
100-kVp markers in model bone	0.17	0.02–0.44	0.07	0.17–0.18
120-kVp markers in prosthesis	0.16	0.01–0.51	0.07	0.15–0.16
100-kVp markers in prosthesis	0.21	0.02–1.06	0.15	0.21–0.22

The effective radiation dose for the model was calculated to be 0.22 milliSievert (mSv) per scan for the 100-kVp scan and 0.36 mSv for scans at 120 kVp.

**Fig. 6:**
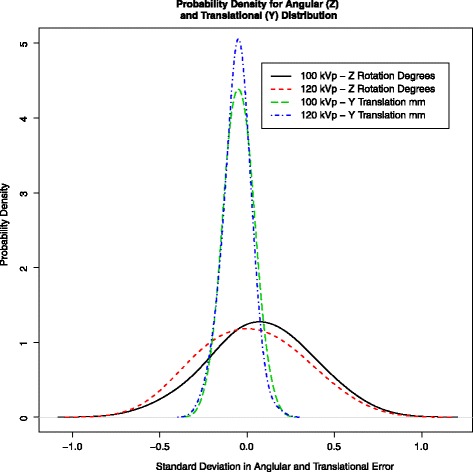
Probability density for the worst rotation angle and translation direction for the first trial from the model. The Gaussian probability density or population frequency of the angular error data though small is more wide spread than that of the translational error

**Fig. 7:**
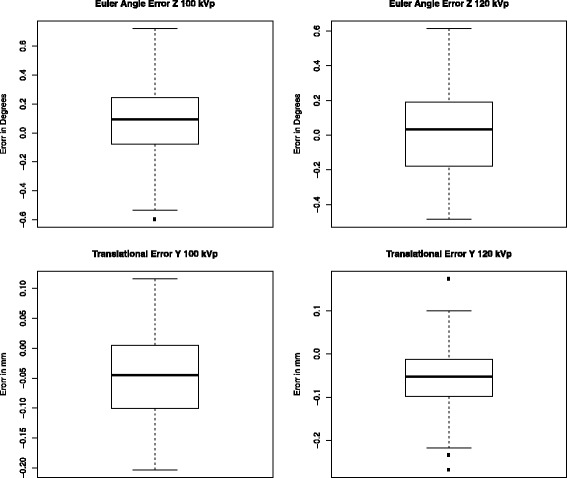
Box plot corresponding to Fig. 6. The median of the error population in each case is close to zero. Although the errors in translation at 120 kVp are within a very small range, there are outliers present

Analysis of relative movement for the model in terms of angular and translational values is given in Table 3. Repeatability and accuracy for measurements of the relative movement is given in Table 4. The ANOVA showed that there were no interactions between the trials as a whole or as a result of the change of kVp (*p* > 0.05). For all six analysis variables, the ANOVA showed there was no significant difference between the studies and the trials, and the residuals were normally distributed around zero. Figure 6 shows the probability density for the *worst* rotation angle and translation direction. The corresponding box plots are shown in Fig. 7.

**Table 3:** Prosthetic movement in the model expressed in six DOF (translation is expressed with respect to the centroid of the prosthetic landmark group) (*n* = 105)

		Rotation (degrees)			Translation (mm)		
		*x*	*y*	*z*	*x*	*y*	*z*
120 kVp, trial 1	Mean	0.02	0.00	0.02	0.00	−0.06	−0.02
	Median	0.01	−0.03	0.03	0.00	0.05	−0.02
	Minimum	−0.45	−0.49	−0.49	−0.11	−0.27	−0.11
	Maximum	0.53	0.59	0.61	0.12	0.17	0.10
	SD	0.17	0.22	0.26	0.05	0.07	0.05
	95 % CI, upper	0.05	0.04	0.07	0.01	−0.04	−0.01
	95 % CI, lower	−0.02	−0.04	−0.03	−0.01	−0.07	−0.03
120 kVp, trial 2	Mean	0.01	0.00	0.02	−0.01	−0.06	−0.02
	Median	0.02	−0.04	0.03	0.00	−0.08	−0.02
	Minimum	−0.46	−0.47	−0.48	−0.11	−0.27	−0.11
	Maximum	0.51	0.56	0.57	0.11	0.17	0.10
	SD	0.17	0.21	0.26	0.05	0.07	0.04
	95 % CI, upper	0.5	0.04	0.7	0.00	−0.04	−0.01
	95 % CI, lower	−0.02	−0.04	−0.03	−0.02	−0.07	−0.03
100 kVp, trial 1	Mean	0.07	0.06	0.08	0.02	−0.05	−0.02
	Median	0.06	0.02	0.09	0.03	−0.04	−0.03
	Minimum	−0.56	−0.59	−0.60	−0.11	−0.20	−0.20
	Maximum	0.61	0.85	0.72	0.15	0.12	0.20
	SD	0.25	0.27	0.27	0.06	0.07	0.08
	95 % CI, upper	0.12	+0.11	0.13	0.04	−0.3	−0.01
	95 % CI, lower	0.02	0.01	0.03	0.01	−0.06	−0.03
100 kVp, trial 2	Mean	0.07	0.06	0.07	0.02	−0.05	−0.02
	Median	0.04	0.03	0.08	0.03	−0.06	−0.2
	Minimum	−0.60	−0.59	−0.62	−0.13	−0.21	−0.19
	Maximum	0.83	0.58	0.72	0.16	0.12	0.20
	SD	0.26	0.27	0.28	0.07	0.07	0.08
	95 % CI, upper	0.12	0.11	0.12	0.04	−0.04	−0.01
	95 % CI, lower	0.02	0.01	0.01	0.01	−0.07	−0.04

**Table 4:** Repeatability and accuracy of measurement of relative movement in the model (*n* = 210)

		Rotation (degrees)			Translation (mm)		
		*x*	*y*	*z*	*x*	*y*	*z*
Repeatability	120 kVp	0.02	0.03	0.03	0.01	0.01	0.01
	100 kVp	0.03	0.04	0.04	0.01	0.01	0.01
Accuracy	120 kVp	0.24	0.30	0.36	0.07	0.13	0.07
	100 kVp	0.37	0.38	0.39	0.09	0.12	0.11

### Patients

**Fig. 8:**
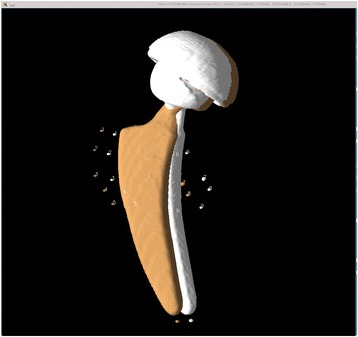
Patient 5 before registration

**Fig. 9:**
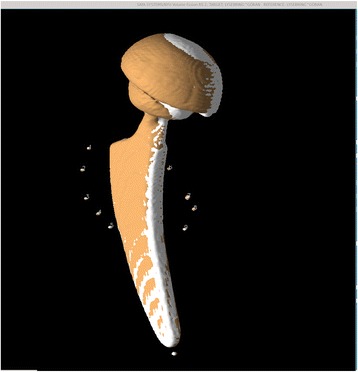
Patient 5 after registration. The tantalum beads are spatially aligned. Only a slight mismatch remains in prosthesis and tip marker

**Fig. 10:**
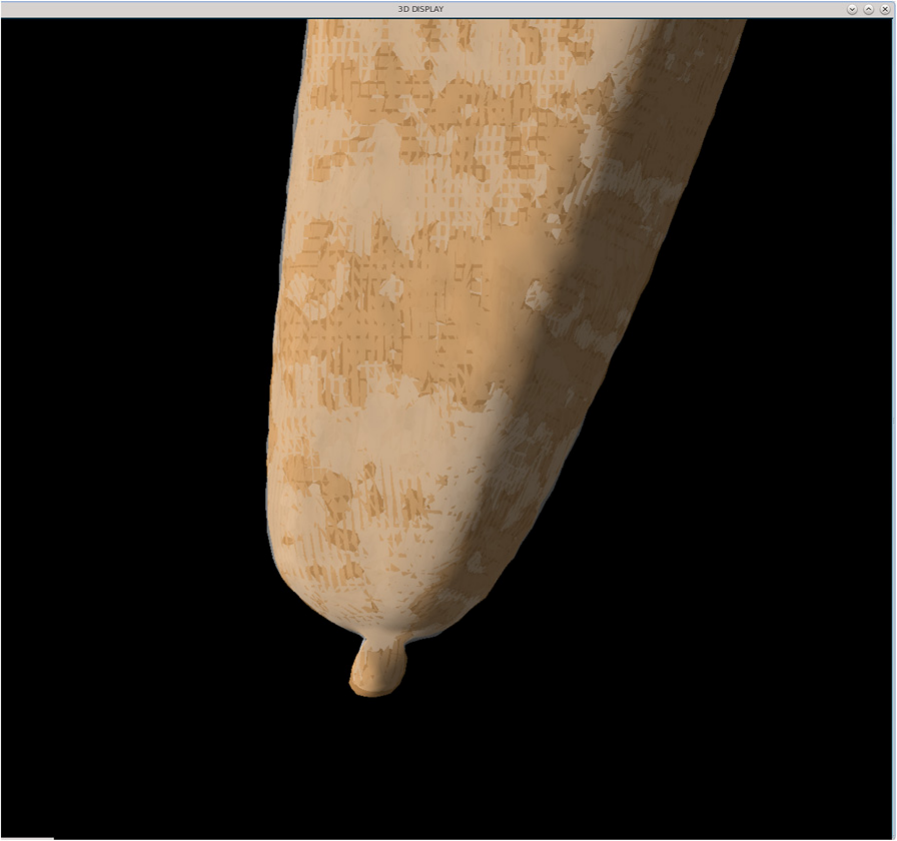
The distal tip of the prosthesis in patient 5 after registration. Semi-transparent display shows that the prosthesis is well registered. A small mismatch of the tantalum bead is seen

In the patients, all tantalum markers corresponding to the bone could be visualized and landmarks designated. There were between eight and 11 markers in the bone for the initial registration. A typical patient volume (patient 5) is illustrated in Figs. 8, 9, and 10. Figure 8 shows the volume before registration. In Fig. 9, the volumes are registered based on the bone landmark sets. Note the overlapping pattern of the bone tantalum beads (Fig. 9) and a slight mismatch in the prosthesis. Figure 10 is a close-up of the registration at the tip of the femoral component in this patient. Note that Figs. 1, 2, 3, 8, 9, and 10 demonstrate how we can visually track and evaluate each major step of the method. Data on relative motion and landmark registration errors are given in Table 5.

**Table 5:** Prosthetic relative movement in the seven patients expressed in six DOF (translation is expressed at the prosthetic landmark group centroid) (*n* = 7)

Patient number	Rotation (degrees)			Translation (mm)			Mean registration error for bone (mm)	Mean registration error for prosthesis (mm)
	*x*	*y*	*z*	*x*	*y*	*z*		
1	0.26	−0.17	−0.06	0. 07	−0.06	−0.01	0.14	0.11
2	−0.10	0. 05	−0.04	−0.01	0.05	0.17	0.24	0.18
3	0.07	−0.12	1.90	0.11	−0.32	0.15	0.14	0.35
4	0.01	0.06	0.52	−0.01	−0.11	−0.06	0.12	0.08
5	−0.06	0.15	0.63	−0.03	0.16	0.11	0.16	0.11
6	−0.04	0.29	−0.37	−0.03	−0.03	−0.03	0.13	0.10
7	0.24	0.07	0.25	−0.03	0.00	−0.09	0.27	0.20

## Discussion

The RSA system incorporates the mean error of rigid body fitting (ME) which reflects the relative motion between individual markers in each segment. This has been used by Markinen et al. [26] to denote the error in registration between different controlled experiments when a rigid body fitting is used. In clinical RSA trials, ME values of 0.10-0.25 mm are typical [27] when using commercially available RSA analysis software (UMRSA—RSA Biomedical, Umeå, Sweden). Laboratory studies show that increasing ME decreases the precision of RSA [28]. Under optimal conditions, the ME numbers can be as small as 0.02-0.05 mm when a phantom is studied with RSA [26]. A recent RSA meta-study has shown that if the proximal migration of the cup in total hip arthropasty is between 0.2 and 1 mm, the patient should be followed for possible revision [29] and that above 1.0 mm, the migration was unacceptable. There is not always a clear relation between migration of the socket and clinical symptoms. In the early stage of the loosening, the socket may often remain “silent” and clinical symptoms appear first when the migration and/or osteolysis is substantial. Especially due to this phenomenon, it is of importance to detect the unstable cups early in order to avoid undesirable bone loss over time. The mean errors found in this study (Table 2) were consistent with detection of cup migration as stated above and are of the same order of magnitude as for clinical RSA trials, indicating that results from CT data could be as precise as results from stereoradiographic data.

When discussing effective radiation dose, it is important to understand that this reflects the potential biological sensitivity of the tissue or organ to the radiation received [30]. It can be seen that for patients 2, 3, and 4 who were examined in 2013 on a more modern CT machine, the effective dose varied between 2.4 and 5.4 mSv (Table 1). These patients were selected at random from the Fitmore study. By contrast, patient 7 who was from the pilot study done in 2010 had an effective radiation dose of 6.6 mSV. The radiation dose in CT is highly dependent on the machine and the protocol used for the examination, as reflected in Table 1. There is no evidence that an effective dose of less than 10 mSv causes harmful medical effects [31]; however, lowering the effective dose without losing information is desirable.

Three dimensional models created from CT have been used to measure kinematics using RSA [32]. Fox and co-workers studied the effect of decreasing CT radiation levels on RSA accuracy at the glenohumeral joint using a dose-length product (DLP) from 750 to 17 mGy/cm and found that accuracy was negligibly affected by the 98 % CT radiation reduction [33]. Several studies on the extremities also show large acceptable effective dose reductions [34–36]. Gurung et al. studied an effective dose reduction in CT of the pelvis using a 16-row CT [37]. Adequate image quality was acquired at an effective dose of 2.2 mSv, for criterion detailed evaluation of acetabulum and iliosacral joint. There is, to our knowledge, no study on dose reduction when imaging radio-opaque markers in the hip. Theoretically, reasonable imaging of the markers should be attainable at significant effective dose reductions, as has been shown in the shoulder [33] and also indicated in this model study.

CT technology continues to improve in resolution, speed, and reduction of effective dose; hence, the effective radiation dose is becoming more and more comparable to that from the exposure from a regular hip X-ray 0.6 mSv [38, 39]. RSA involves two X-rays. Valstar et al. [6] state (with qualifications) that “The radiation doses for most standard RSA examinations have been evaluated, and have proven to be lower than for the corresponding conventional examinations.” Some values for actual effective dose in RSA have been tabulated in Valstar’s thesis [40]. However, in practice, RSA examinations involve frequent additional retakes.

In contrast, CT scans are easily acquired and the examination can be performed on any modern CT unit. The acquisition is fast and unlike RSA [6], patient positioning is not vital, since the CT volume can be transformed into an arbitrary spatial orientation. There are several potential benefits from using CT as opposed to marker-based RSA: (1) minimizing the risk of examination exclusion due to obscured markers, which is common in RSA [41]; (2) greatly speeding up the marking process, since marker identification becomes trivial when utilizing powerful, interactive 2D and 3D visualization tools applied to the CT volume data; and (3) readily enabling 3D evaluation of marker configuration and distribution. In addition to reporting the relative motion numerically, the CT method gives immediate visual feedback both in 2D and 3D, with volumes displayed either side-by-side or fused. Therefore, the quality of the registration, in this case based on the markers attached to the bone, as well as the relative movement, can be visually evaluated. Any point in these volumes can be accessed and designated, so it is possible to study the relative movement at any location. Additionally, non-marker-based points can be added if necessary for performing the transformations, as was done in two of the patients. Further, the use of the landmark-based SVD minimizes the effect of misplaced landmarks. This was demonstrated in the model prosthetic landmark set, marker number 5. The removal of landmark 5 from the set, did *not* affect the values of the Euler angles and translation errors associated with the trials. Thus, there is an apparent potential for practical use of this method in evaluation of primary and secondary stability of the orthopedic implants especially when introducing new designs or modifying existing implants. This has also been studied by Derwin et al. for the rotator cuff [42].

Disadvantages of the proposed method are that it is new and relatively untested and has not been validated as much as RSA. Additionally, it requires user interaction which could vary from one operator to another. To our knowledge, there is no commercially available CT RSA analysis suite at present. Although this method has only been applied to seven patients in this feasibility study, a further study, applied to 45 patients (48 hip implants), comparing CT and RSA performed on the same day has been completed and submitted.

In previous publications, we have shown that RSA data could be retrospectively registered to, and visualized in, CT volumes [43, 44]. We have conducted a phantom study where the repeatability and accuracy of the CT method is directly compared using double RSA and CT studies taken at the same time and have shown that the scans, if applied to a patient, would give an effective dose of 0.4 mSv. The effective dose has also been reduced even further in an ongoing porcine model study.

## Conclusions

The accuracy and repeatability for the model studies were comparable to those reported for similar RSA studies. The mean errors of rigid body fitting in both the model and patient studies were comparable to the errors of 0.10-0.25 mm reported in typical clinical RSA trials. In the seven patients, this technique was able to evaluate two patients who would have been unable to be evaluated if only RSA imaging and analysis were used. Thus, the analysis of both the model and the seven patients showed that the proposed technique can be used to evaluate patients with tantalum beads, thus avoiding the inability to evaluate these patients over time due to the lack of facilities for doing stereoradiographs or marker movement. Further, the effective dose associated with CT is decreasing.

## Abbreviations

CT: computed tomography
DPL: dose-length product
kV: kilovoltage
kVp: kilovoltage peak
mAs: milliampere-seconds
ME: mean error of rigid body fitting
mSv: milliSievert
RSA: radiostereometric analysis
SD: standard deviation
SEMAR: single-energy metal artifact reduction
SVD: singular value decomposition
